# Response of Plant Community Characteristics and Soil Factors to Topographic Variations in Alpine Grasslands

**DOI:** 10.3390/plants14010063

**Published:** 2024-12-28

**Authors:** Qinyang Liang, Jinmei Zhao, Zixin Wang, Xingyi Wang, Dianxia Fu, Xiaogang Li

**Affiliations:** 1 College of Forestry, Gansu Agricultural University, Lanzhou 730070, China; lqy0191@163.com (Q.L.); wzxw9476@163.com (Z.W.); wxyhylys@163.com (X.W.); 2 Gansu Liancheng National Nature Reserve Administration, Lanzhou 730300, China; fudx0910@163.com (D.F.); lxgd2009@163.com (X.L.)

**Keywords:** alpine grassland, slope aspect, slope position, plant community, soil properties, plant–soil relationship

## Abstract

Topography has an important influence on plant–soil relationships. However, research on plant–soil relationships in alpine grassland at the slope aspect and slope position scales is currently inadequate. In this paper, based on the topographic and geomorphological characteristics of the study area, alpine grassland with typical slope aspect and slope position conditions was selected as the research object. Through field investigations and laboratory research to reveal how the characteristics of the alpine grassland plant community and soil factors respond to changes in topography. The results show: Slope aspect and slope position changes significantly affect alpine grassland plant communities and soil properties. In terms of the dominant species in plant communities, the sunny slopes were dominated by Poaceae and the shady slopes were dominated by Polygonaceae. Plant community characterization variables showed a decreasing trend from shady to sunny slopes and bottom to top. The soil factors showed significant differences among the six types of topography (*p* < 0.05), and the magnitude order in different slope aspects and positions was basically shady slope > sunny slope and bottom > middle and top. Correlation analysis showed that there were good correlations between soil organic carbon (SOC), soil water content (SWC), total nitrogen (TN), pH, and plant community characteristics in alpine grassland. In addition, redundancy analyses (RDA) indicated that the divergence in plant community characteristics was primarily driven by the change difference in SOC along topographic gradients. Our findings may provide a scientific basis for the restoration and utilization of alpine grassland vegetation and the evaluation of the ecological environment in this region.

## 1. Introduction

Alpine grassland, which is a widely distributed vegetation type in the eastern Qilian Mountains of China, is adapted to the unique environment shaped by the plateau’s uplift and prolonged low temperatures [1]. Alpine grassland serves not only as the material foundation for the development of plateau livestock husbandry and the economic advancement of local ethnic groups but also plays an irreplaceable role in regulating groundwater and surface water and in mitigating natural disasters, thereby stabilizing ecological services [2]. The mountainous terrain typically characterizes the distribution of alpine grasslands [3]. As unique habitat islands, the environmental heterogeneity induced by topographic changes further drives differences in the overall evolution of plants and the environment [4]. These differences impact the interrelationships between plants and soils, leading to complex interactions during their evolutionary processes and reflecting the efficiency of resource allocation and utilization under varying topographic conditions. The alpine grassland ecosystem has the characteristics of vulnerability and instability due to its “high” and “cold” natural environment [5], which features a unique ecological hydrological process sensitive to climate change. However, the underlying surface of the alpine mountainous area is complex, and the large water and heat gradient, along with the varied and dynamic terrain, causes differences in water and heat patterns [6], intensifying the interaction between plant and soil. Studies focusing on how topographic factors influence the spatial heterogeneity of soils and plants remain insufficient in alpine grassland areas. To better understand the spatial heterogeneity of soils and plants under complex topographic conditions and to identify areas of high conservation value for ecological restoration, this study examines the changes in soil and plant properties of grassland ecosystems on the slope aspect and slope position gradients in alpine mountains. The results of the study will not only provide a theoretical basis for subsequent scientific decision-making and management but also offer robust support for addressing the challenges of ecological conservation in the context of global climate change.

Within the same elevation band, slope aspect and slope position are important topographic factors influencing soil and vegetation patterns at small scales in mountainous areas [7] and are usually closely related to habitat conditions [8]. The morphological structure of different topographies redistributes solar radiation, precipitation, litter storage, and mineral elements in local habitats. It also impacts the spatial arrangement of internal soil temperature, water, and nutrients by adjusting precipitation infiltration and soil evaporation, thereby altering plant distribution. Conversely, plants affect the accumulation and dispersion of soil nutrients, and the interaction between these factors creates unique niches in diverse topographies [7,9,10]. As one of the most important mountain environmental factors, pronounced changes in slope aspect habitats lead to the creation of localized specific climates, which in turn directly or indirectly affect plants, soils, and the relationship between them [11]. Water–heat combinations between different slope orientations, and the degree of soil fertility, result in significant changes in plant community species diversity, productivity, and community succession [7,12]. In addition, the slope aspect also influences the intensity of solar radiation, with shaded slopes receiving less solar radiation and lower temperatures for shade-loving plants, while sunny slopes are well-lit and form plant communities dominated by drought-tolerant plants [13]. Therefore, the aspect is a complex topographical dimension that affects plant and soil. The geomorphological properties of different slope sites in alpine mountains vary, with both the top and middle slopes being relatively steep and undulating, whereas the bottom slope is mostly gentle. Research indicates that slope position significantly impacts soil moisture, fertility, and plant light conditions. In terms of soil moisture, steep slopes may create harsher environments for water use, and the degree of soil moisture variability tends to positively correlate with topographic heterogeneity [14]. Following rainfall, water moves to the lower parts of the slope via surface runoff and loamy intermediate flow due to gravity, resulting in uneven water distribution, which affects soil qualities and plant characteristics [15]. In terms of soil fertility, slope is the basic module that affects soil nutrients; differences in soil structure between slope positions lead to variations in soil bulk density, saturated hydraulic conductivity, and disintegration rate, thereby altering soil fertility [16]. In terms of plant light conditions, slope position is regarded as a key factor influencing plant distribution and growth; plants on upper and lower slopes receive more light, providing a solid foundation for growth and enhancing the soil consolidation ability of plant roots, which promotes material circulation and can effectively improve soil stability and nutrient content [17]. Different dimensions of topography have their emphases; each dimension does not exist in isolation but interacts together to form a complex impact on the plant–soil relationship [18]. Slope aspect and position remodel soil-forming processes and plant growth at small scales, and the superimposed effects of such influences lead to spatial heterogeneity of soil and plant properties within the same elevation band, an accurate understanding of which may explain environmental filtering effects that cannot be accounted for in large-scale studies [19].

As the fundamental topographic unit for managing grassland ecosystems in alpine mountainous areas, small-scale mountains eliminate the climatic differences caused by varying geographical regions, making an ideal place for studying the relationship between topography–plant–soil under the interplay of slope aspect and position in alpine mountainous areas. Consequently, this paper focuses on the typical slope aspect and position of alpine grassland in Tulugou National Forest Park within the Gansu Liancheng National Nature Reserve. By combining soil environmental factors from the sample plot, this study aims to elucidate two scientific questions: (1) What are the patterns and causes of differentiation in plant community characteristics and soil factors across different slope aspects and positions in alpine grasslands? (2) What are the links between plant community characteristics and soil factors under slope aspect and position gradients, and which soil factors can effectively explain the differentiation of plant community features? The objective is to lay a theoretical foundation for the protection of grassland ecosystems and the restoration of plant degradation in fragile alpine mountains, as well as to contribute positively to the national strategy for ecological protection and high-quality development in the Yellow River Basin.

## 2. Materials and Methods

### 2.1. Study Site Description

The study area is situated in Tulugou National Forest Park within the Gansu Liancheng National Nature Reserve, located west of Yongdeng County, Gansu Province, China (36°43′38″ N, 102°36′53″ E). It is located in the transition area between the Qinghai–Tibet Plateau, Qilian Mountains, Loess Plateau, and Longxi subsidence basin. The elevation ranges from 2000 to 3600 m, and the terrain is complex and varied; the elevation difference is large (300 to 700 m). This area falls within the temperate semi-arid climate zone of the Qilian Mountains–northern Longzhong, characterized by distinct temperate continental climate features. Over the period from 2019 to 2023, the alpine grassland distribution area recorded an average temperature of 7.4 °C, an annual average maximum temperature of 16.5 °C, an annual average minimum temperature of −1.9 °C, and an annual average precipitation of 419 mm, predominantly occurring from June to September and accounting for 60% of the annual total. The annual average evaporation rate was 1542 mm, the annual sunshine duration was 2655.2 h, the annual total solar radiation was 469 kJ·cm^−1^, and the frost-free period ranged from 125 to 135 days. The soil layer in the research region is relatively thin, measuring 40 to 80 cm, and the soil type primarily consists of dark, felty soils.

### 2.2. Experimental Design

**Figure 1:**
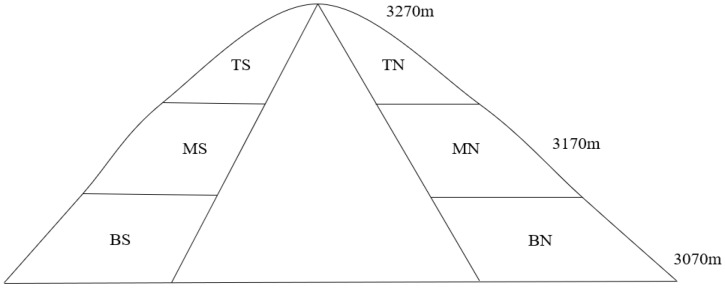
Sample plots layout map.

The experiment was conducted in early August 2023. The sample plots are far away from the frequent human activities and grazing areas, reducing the interference as far as possible to select the representative alpine grassland close to the natural state as the research object. The selection of sample plots was based on the topography and geomorphological features of the research region, adhering to the principle of consistent elevation for the same slope position. The plots were categorized based on two factors: slope aspect and slope position. Slope aspects were divided into two categories: sunny slope (south-facing) and shady slope (north-facing). Based on elevation, the different slope aspects were further divided into three types: bottom, middle, and top, resulting in a total of six types of sample plots (Figure 1): bottom of the sunny slope (BS), middle of the sunny slope (MS), top of the sunny slope (TS), bottom of the shady slope (BN), middle of the shady slope (MN), and top of the shady slope (TN). In each sample plot, three sample quadrats of 1 m × 1 m (area) were laid out diagonally. Initially, the latitude, longitude, elevation, and slope information of the quadrats were recorded using GPS. Subsequently, the plant species, height, coverage, number of plants, and biomass of the alpine grassland within these quadrats were surveyed, and soil samples were collected using soil drills. Table 1 provides a detailed overview of the sample plots.

### 2.3. Plant Biomass Collection and Measurement

Aboveground biomass (AGB) was collected using the direct harvest method. Three groups of sample squares with representative plants and similar growth were laid out in each sample plot, using the size of a 25 cm × 25 cm sample box for circular sampling. The upper part of the plant inside the sample box was cut at ground level with scissors, placed into an envelope bag, clearly labeled, and transported back to the laboratory. It was initially killed and processed in a drying oven at 105 °C for 30 min, then dried to a constant weight at 65 °C for 12 h before being weighed. In the sample square where aboveground biomass was collected, belowground biomass (BGB) was gathered using the stratified excavation method. Five layers (0–10, 10–20, 20–30, 30–40, 40–50 cm) of soil columns with a volume of 25 cm (length) × 25 cm (width) × 10 cm (height) were excavated in each sample square, with each layer being excavated three times. The excavated root samples were placed into nylon bags for multiple rinsings to remove soil, gravel, and debris, then transferred to envelope bags, labeled, and dried in an oven to a constant weight (65 °C for 12 h) before weighing. The formula for calculating biomass is as follows: Plant Biomass (g·m^−2^) = Plant Dry Mass (g)/Sample Area (m^2^) [20].

### 2.4. Plant Species Diversity Index Calculation

The alpha diversity index was employed to calculate the diversity index of the alpine grassland plant community. The species importance value formula used in this study is Important Value (%) = (Relative Coverage + Relative Height + Relative Frequency)/3. To characterize the alpha diversity of species, the following indices were selected: Margalef (*Ma*) species richness index, Simpson (*P*) dominance index, Shannon–Wiener (*H*′) diversity index, and Pielou (*E*) evenness index. The calculation methods for each index are based on previous studies [21]. The formulas are as follows:

(1) $$\begin{array}{c} Ma=\frac{\left(S-1\right)}{\ln N} \end{array}$$

(2) $$\begin{array}{c} P=1-\sum {P_{i}}^{2} \end{array}$$

(3) $$\begin{array}{c} H^{\prime }=-\sum_{i=1}^{S}P_{i}\ln P_{i} \end{array}$$

(4) $$\begin{array}{c} E=\frac{H}{\ln S} \end{array}$$

In the above formula, *S* is the number of species, *N* is the total number of individuals across all species, and *Pi* is the important value of the *i*-th species.

### 2.5. Soil Sample Collection and Measurement

The layout of the soil sampling squares mirrored that of the plant sampling squares. In each survey sample plot, an area with minimal human interference and representative plant structure and soil was selected. Soil samples were collected using a soil drill with stratification at depths of 0–10, 10–20, and 20–30 cm, with three replications per stratum following an “S” pattern. Fresh soil samples from the same squares and layer were sieved through a 2 mm sieve, debris such as plant roots and stones was removed, and the soil was divided into two parts: one part was refrigerated at 4 °C to determine soil water content and nitrate–ammonium nitrogen content, while the other part was air-dried in a ventilated, dark place; after further grinding, the soil organic carbon, total nitrogen, total phosphorus, available phosphorus, and pH content were determined.

The soil water content (SWC) was determined using the drying weighing method (105 °C, 12 h). Soil organic carbon (SOC) was measured by the potassium dichromate volumetric method with external heating. Soil total nitrogen (TN) was assessed using the Kjeldahl method (K9840 Automatic Kjeldahl Nitrogen Analyzer, Jinan, China). Soil total phosphorus (TP) was determined by the molybdenum antimony colorimetric method. Soil nitrate nitrogen (NO_3_^−^-N) and ammonium nitrogen (NH_4_^+^-N) were extracted with a 2 mol/L KCI solution and measured using a nitrogen analyzer (K9840 Automatic Kjeldahl Nitrogen Analyzer, Jinan, China). Soil available phosphorus (AP) was determined by the sodium bicarbonate extraction–molybdenum antimony colorimetric method. The soil pH value was measured by the potentiometric method (water–soil ratio of 5:1) (MP-551 pH/Conductivity Meter, Shanghai, China). Each soil sample was measured three times to ensure accuracy. The detailed methodologies for each index determination can be found in the relevant literature [22].

### 2.6. Data Analysis

In order to assess differences between plant community traits and soil environmental factors under various slope aspects and positions, the standardized plant and soil data were subjected to one-way ANOVA using SPSS 26.0 software (SPSS Inc., Chicago, IL, USA), using *p* < 0.05 as the significant level standard. When the variance was homogeneous, Duncan’s new multiple range method was employed; when the variance was heterogeneous, differences were assessed using Welch’s test. If the *F*-test was significant, the Least Significant Difference (LSD) test was conducted to compare means. A two-factor variance analysis (two-way ANOVA) was utilized to examine the impacts of slope aspect, slope position, and their interaction on plant community characteristics and soil factors, as well as to identify the main variation factors. Pearson correlation analysis was used to evaluate the relationships between plant community traits and soil variables. Detrended correspondence analysis (DCA) with a maximum gradient of 0.2 on the four sorting axes was conducted using Canoco 5.0 software; subsequently, redundancy analysis (RDA) was applied to analyze the key soil driving elements influencing plant community traits. Drawing tools used Origin Pro 2021. Results were presented as mean ± standard deviation.

## 3. Results

### 3.1. Plant Community Characteristics

#### 3.1.1. Characteristic Changes in Plant Distribution

The vegetation structure of alpine grassland in this study was of pure herbaceous type. In the six types of sample plots, total plant cover was largest in BN and smallest in TS, with the following size order: BN (98%) > MN (96%) > BS (95%) > MS (93%) > TN (92%) > TS (90%); the height of the grass layer was largest in BN and smallest in TS, with the following size order: BN (12.38 cm) > BS (11.49 cm) > MN (10.57 cm) > MS (10.27 cm) > TN (9.69 cm) > TS (8.78 cm) (Table 2).

The total number of families of alpine grassland plants in the study area ranged from 8 to 14, the total number of genera ranged from 16 to 23, and the total number of species ranged from 20 to 27 (Table 2). The number of families, genera, and species was higher on shady slopes than on sunny slopes and higher at the bottom than at the middle and top of the slope. The composition of plant communities was dominated by seven families in total, including Poaceae, Leguminosae, Compositae, Rosaceae, Cyperaceae, Polygonaceae, and Ranunculaceae, which accounted for 68.46% of the total number of species in the six types of topography. Affected by the mountainous terrain conditions, the distribution of plants in alpine grasslands of different topographies also varied, in which Poaceae, Cyperaceae, and Polygonaceae were distributed in six types of topographies, whereas Leguminosae was not distributed in BS and MS, Compositae was not distributed in MS and TS, Rosaceae was not distributed in MN, and Ranunculaceae was also not distributed in TN.

In terms of dominant species (Table 2), sunny slopes are dominated by mesic drought-tolerant Poaceae, and the dominant species is *Elymus dahuricus*; shady slopes are dominated by mesic shade-tolerant Polygonaceae, and the dominant species is *Polygonum viviparum*. Influenced by the mountainous terrain conditions, the dominant species of different slope aspect and slope position alpine grassland vegetation also varied, with BS dominated by *Elymus dahuricus* and *Kobresia humilis*, MS dominated by *Elymus dahuricus*, BN dominated by *Elymus dahuricus* and *Polygonum viviparum*, MN dominated by *Polygonum viviparum* and *Kobresia humilis*, and both TS and TN dominated by *Polygonum viviparum*.

#### 3.1.2. Plant Community α Diversity

**Figure 2:**
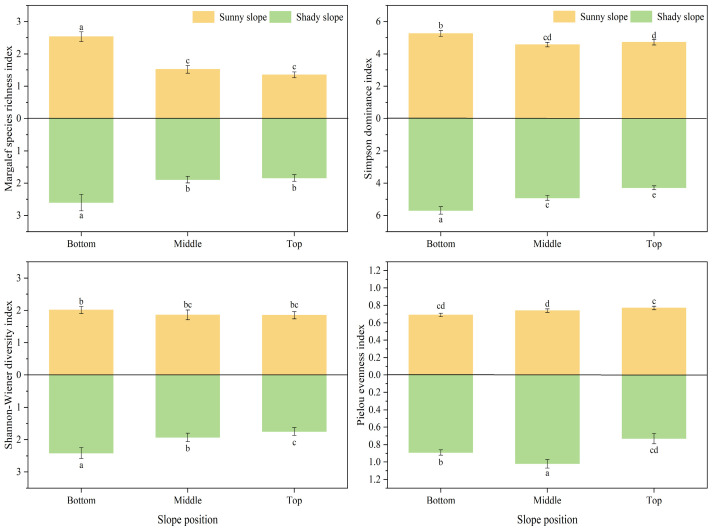
Difference in α biodiversity index in different slope aspects and slope positions. Note: differences between topographies at the 0.05 level are indicated by lowercase letters.

The Margalef (*Ma*), Simpson (*P*), Shannon–Wiener (*H*′), and Pielou (*E*) indices for the six types of topographies varied between 1.35 and 2.60, 4.28 and 5.69, 1.75 and 2.41, and 0.69 and 1.01, respectively (Figure 2). Across different slope aspects, the indices on the shady slope were consistently higher than those on the sunny slope, with increases of 17.22% for *Ma*, 2.27% for *P*, 6.47% for *H*′, and 20.00% for *E*, compared to the sunny slope. As the slope position rose, the *Ma* and *H*′ indices showed a decreasing trend; the *P* index exhibited a “V” shaped pattern on the sunny slope and a decreasing trend on the shady slope; the *E* index increased on the sunny slope and displayed a single-peak pattern of initially increasing then decreasing on the shady slope. The differences in α diversity indices across various topographies were statistically significant (*p* < 0.05), indicating that slope aspect and position significantly influence plant species composition and, consequently, plant community characteristics.

#### 3.1.3. Plant Community Biomass

**Figure 3:**
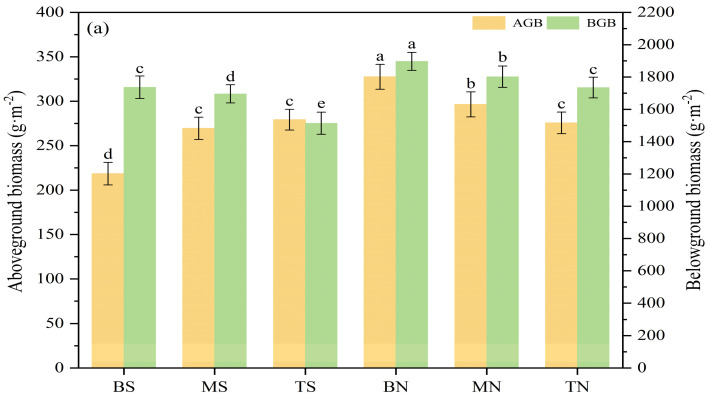

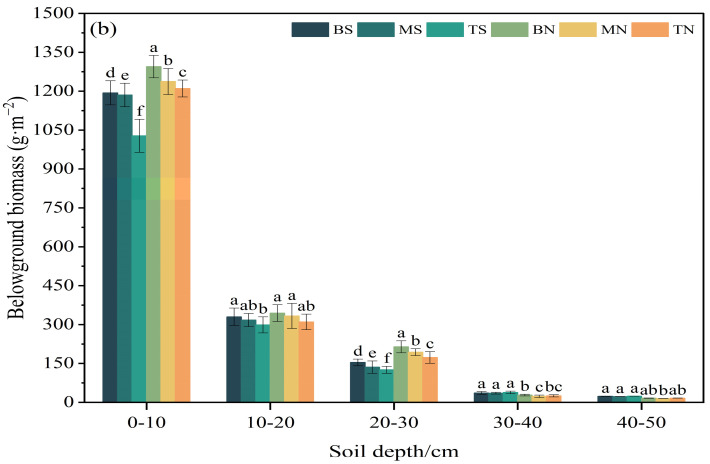
(**a**) Biomass characteristics of different topographies. Note: differences between topographies at the 0.05 level are indicated by lowercase letters. (**b**) Belowground biomass in 0–50 cm soil layers. Note: differences within the same soil depth but different topographies are represented by lowercase letters (*p* < 0.05). AGB and BGB represent aboveground biomass and belowground biomass, respectively.

Based on the results of the analysis of variance of biomass in different topographies (Figure 3a), it can be seen that in terms of aboveground biomass, shady slopes (299.89 g·m^−2^) were higher than sunny slopes (255.74 g·m^−2^). As the slope position increased, the sunny slope showed an increasing trend, while the shady slope exhibited a significant decreasing trend (*p* < 0.05). The aboveground biomass peaked at BN (327.56 g·m^−2^) and reached its valley value at BS (218.52 g·m^−2^), with a difference of 49.89%. Regarding belowground biomass, shady slopes (1811.25 g·m^−2^) were higher than sunny slopes (1648.86 g·m^−2^), with a significant decreasing trend with increasing slope position for both sunny and shady slopes (*p* < 0.05). Belowground biomass peaked at BN (1896.97 g·m^−2^) and reached the valley value at TS (1514.05 g·m^−2^) with a difference of 25.29%.

In the study area, the belowground biomass of all six types of topographies, alpine grasslands, gradually decreased with increasing soil depth; there were relatively significant differences in the belowground biomass of the 0–50 cm soil layer among the various topographies (*p* < 0.05), as shown in Figure 3b. Belowground biomass in the 0–10 cm soil layer constituted 68.87% of the total belowground biomass found in the 0–50 cm soil layer. The belowground biomass in the 0–10 cm soil layer was 3.69, 7.18, 38.41, and 61.26 times greater than that in the 10–20, 20–30, 30–40, and 40–50 cm soil layers, respectively. Remarkably, 86.16% of the total biomass was located underground, with 59.34% of it concentrated in the surface soil (0–10 cm).

#### 3.1.4. Effects of Slope Aspect and Slope Position on Plant Community Characteristics

The two-way analysis of variance (Table 3) revealed significant effects of the slope aspect on GH, *Ma*, and *E* (*p* < 0.01), as well as on *H*′, AGB, and BGB (*p* < 0.05). Slope position significantly influenced *Ma*, *H*′, and BGB (*p* < 0.01), as well as TC, GH, *P*, and AGB (*p* < 0.05). The interaction between slope aspect and position significantly affected AGB and BGB (*p* < 0.01), as well as *Ma* and *H*′ (*p* < 0.05). The *F* values indicated that the slope aspect had a greater impact on *E*, AGB, and BGB than the slope position, whereas slope position affects TC, GH, *Ma*, *P*, and *H*′ to a greater extent than the slope aspect.

### 3.2. Soil Factor

#### 3.2.1. Soil Water Content

**Figure 4:**
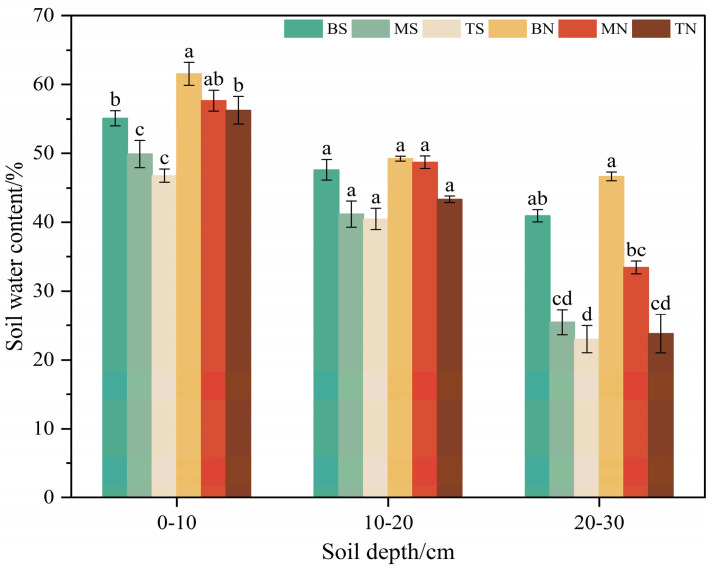
Soil water content in different topographies. Note: Differences within the same soil depth but different topographies are represented by lowercase letters (*p* < 0.05), the same as below.

Figure 4 illustrates that at the 0–10 cm soil depth, BS (55.11%) and BN (61.54%) exhibited higher soil water content compared to other slope positions within the same slope aspect. MS and TS showed reductions of 9.45% and 15.15%, respectively, compared to BS, while MN and TN showed decreases of 6.32% and 8.56%, respectively, compared to BN. The differences in soil water content across various topographies were significant (*p* < 0.05). At the 10–20 cm soil depth, there was no significant variation in soil water content across different topographies (*p* > 0.05), indicating minimal influence of topographical changes on soil water content at this layer. In the 20–30 cm soil depth, BS (40.93%) and BN (46.66%) had significantly higher soil water content compared to other slope positions within the same slope aspect (*p* < 0.05), with BS having 1.61 and 1.78 times the soil water content of MS and TS, respectively, and BN having 1.39 and 1.96 times that of MN and TN. Comprehensive comparisons show that soil water content followed the pattern of shady slope > sunny slope and bottom > middle > top.

#### 3.2.2. Soil Total Nutrients

**Figure 5:**
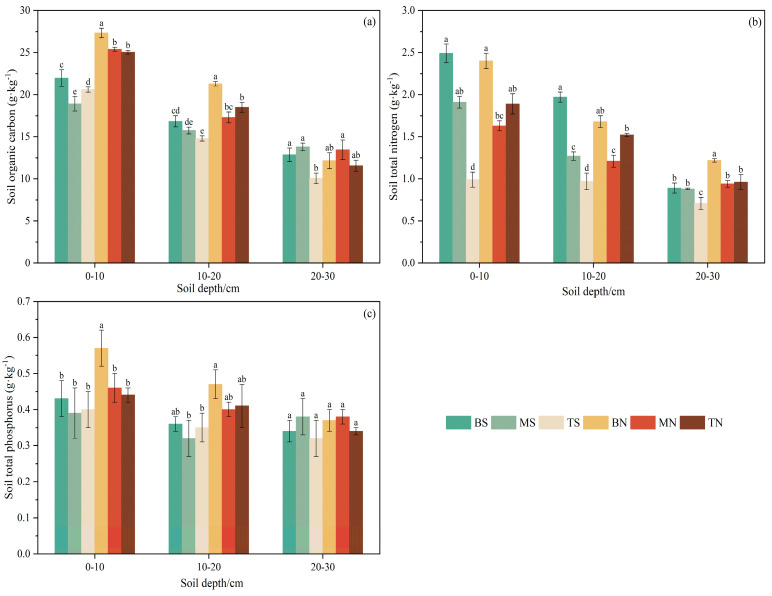
(**a**–**c**) Soil total nutrient content in different topographies.

Figure 5a reveals that at the 0–20 cm soil depth, BS (21.97, 16.84 g·kg^−1^) and BN (27.34, 21.29 g·kg^−1^) had significantly higher soil organic carbon content than other slope positions within the same slope aspect (*p* < 0.05). At the 20–30 cm depth, soil organic carbon content in different slope positions on both sunny and shady slopes exhibited a single-peak trend of increasing and then decreasing, with significant differences only between TS and BS, MS, and MN (*p* < 0.05). Overall, soil organic carbon content was higher on the shaded slope than on the sunny slope in the 0–20 cm soil horizons, and both were enriched at the bottom of the shady and sunny slopes, although the organic carbon content in the 20–30 cm soil depth was slightly higher on the sunny slope than the shady slope.

Figure 5b shows that in the 0–30 cm soil horizons of the sunny slope, soil total nitrogen content decreased with increasing slope position, peaking at BS (2.49, 1.97, 0.89 g·kg^−1^) and lowest at TS (0.99, 0.97, 0.71 g·kg^−1^), with significant differences (*p* < 0.05). On the shady slope, soil total nitrogen content followed a “V” pattern with the rise in slope position, peaking at BN (2.40, 1.68, 1.22 g·kg^−1^) and lowest at MN (1.63, 1.21, 0.94 g·kg^−1^), with significant differences (*p* < 0.05).

Figure 5c indicates that in the 0–10 cm soil depth, BN had significantly higher soil total phosphorus content compared to other topographies (*p* < 0.05), with values 1.33, 1.46, 1.43, 1.24, and 1.30 times those of BS, MS, TS, MN, and TN, respectively. In the 10–20 cm soil horizons, the soil total phosphorus content was in descending order from BN, TN, MN, BS, and TS to MS, with only BN being significantly different from MS and TS (*p* < 0.05). In the 20–30 cm soil horizons, there was no significant variation in soil total phosphorus content across different topographies (*p* > 0.05), indicating that changes in topographies had little influence on soil total phosphorus content in this layer.

#### 3.2.3. Soil Available Nutrients

**Figure 6:**
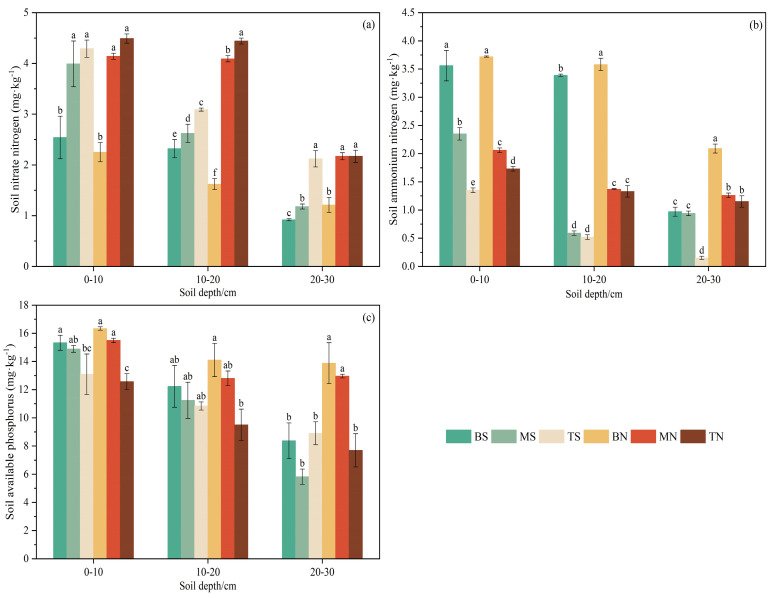
Soil available nutrient content in different topographies.

The analysis of soil available nutrient content in alpine grassland with different slope aspects and slope positions revealed that (Figure 6), with the increase in slope position, the contents of soil ammonium nitrogen and available phosphorus on both sunny and shady slopes exhibited a synergistic downward trend (bottom > middle > top) across different soil depths. Conversely, soil nitrate nitrogen content increased with the rise in slope position (bottom < middle < top). This pattern may be attributed to higher soil compaction at the bottom due to gravity, resulting in poorer soil aeration and less effective nitrogen oxidation compared to the top areas. Across the 0–30 cm soil depth, soil available nutrient content steadily dropped from the surface to the bottom, with the overall trend being that shady slopes are higher than sunny slopes. Significant variations in soil available nutrient content across different topographies (*p* < 0.05) indicate a substantial impact on its distribution.

#### 3.2.4. Soil pH

The soil pH across the six types of topographies in the study area ranged from 5.52 to 5.95, averaging slightly acidic at 5.7 (Table 4). In the 0–10 cm soil horizons, the pH on both sunny and shady slopes increased with rising slope position, with the pH at TS (5.91) being significantly higher than at BS (5.52) and BN (5.53) (*p* < 0.05). In the 10–30 cm soil horizons, the pH on the sunny slope displayed an unimodal pattern of initially increasing and then decreasing with elevation, while on the shady slope, pH consistently rose with increasing slope position. Notably, the soil pH at TN (5.93) in the 10–20 cm soil horizons was significantly higher than at BS (5.66) (*p* < 0.05), and the pH at TN (5.95) in the 20–30 cm soil horizons was significantly higher than at BN (5.73) (*p* < 0.05), with no significant differences between other topographies.

#### 3.2.5. Effects of Slope Aspect and Slope Position on Soil Factors

The two-way analysis of variance (Table 5) showed that the slope aspect significantly influenced SWC (*p* < 0.01), as well as SOC and TN (*p* < 0.05). Slope position significantly influenced SWC and SOC (*p* < 0.01), as well as TN, TP, NO_3_^−^-N, and NH_4_^+^-N (*p* < 0.05). There was a highly significant interaction between the slope aspect and position on NO_3_^−^-N, NH_4_^+^-N, and AP (*p* < 0.01). The F values indicated that the slope aspect had a greater impact on SOC and TP than the slope position, while the slope position had a more pronounced effect on SWC, TN, NO_3_^−^-N, NH_4_^+^-N, AP, and pH than the slope aspect.

### 3.3. Correlation Between Plant Community Characteristics and Soil Factors

**Figure 7:**
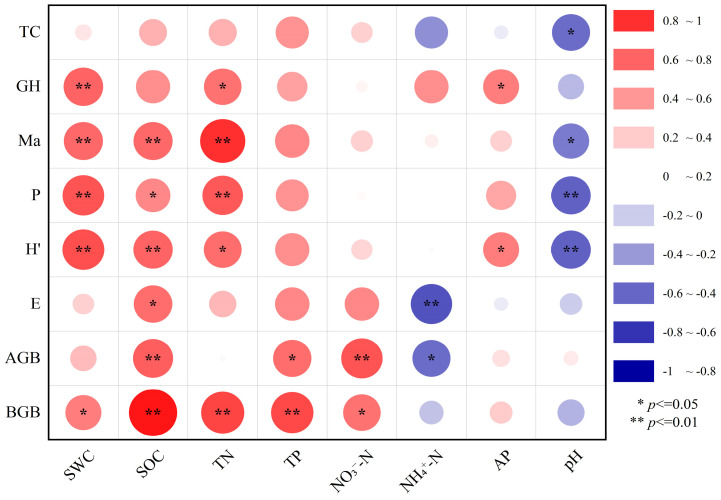
Relationship between plant community characteristics and soil factors.

Potential links between plant community characteristics and soil factors revealed by correlation heat maps (Figure 7). The research findings: SWC and GH, *Ma*, *P*, *H*′, SOC and *Ma*, *H*′, AGB, BGB, TN and *Ma*, *P*, BGB, TP and BGB, NO_3_^−^-N and AGB were highly significant positive correlations (*p* < 0.01). NH_4_^+^-N and *E*, pH, and *P*, *H*′ were highly significant negative correlations (*p* < 0.01). There were significant correlations between SWC and BGB, SOC and *P*, *E*, TN and GH, *H*′, TP and AGB, NO_3_^−^-N and BGB, NH_4_^+^-N and AGB, AP and GH, *H*′, pH and TC, and *Ma*. Overall, SWC, SOC, TN, and pH are key soil factors impacting the characteristics of alpine grassland plant communities.

### 3.4. Plant Community Characteristics and Soil Factor RDA Analysis

Redundancy analysis (RDA) is a linear model that merges correspondence analysis with multiple regression to rank variables, providing a visual representation of the influence of soil factors on plant community characteristics. The correlation coefficients between plant community characteristics and soil factors for the first and second axes are 0.9908 and 0.9140, respectively, and the ranking results are reliable (Table 6). The explanatory powers of RDA1 and RDA2 are 94.30% and 3.27%, respectively, cumulatively explaining 97.56% of the variance, with a cumulative explanatory rate of 99.97%. This indicates the critical role of the first and second axes in delineating the relationship between plant community characteristics and soil factors.

**Figure 8:**
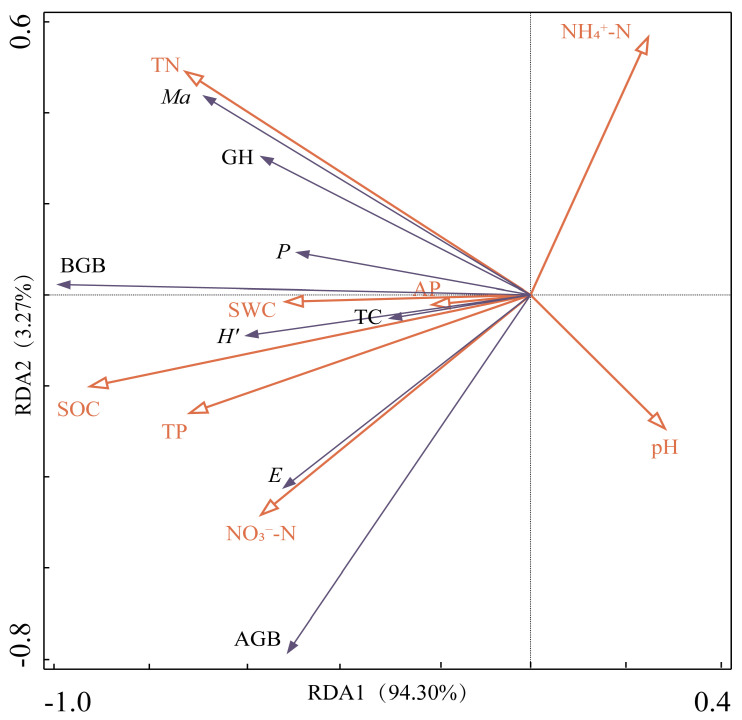
Plant community characteristics and soil factors RDA ordination diagram. Note: plant community characteristics are indicated by blue solid arrows, and soil environmental factors are indicated by red hollow arrows.

The Monte Carlo test (Table 7) and RDA ordination diagram (Figure 8) identified SOC as the primary driver influencing the plant community characteristics of alpine grassland, followed by TN; the explanation rates for the variation in plant community characteristics were 80.6% and 7.5% (*p* < 0.05), with contribution rates of 82.6% and 7.7%, and a combined contribution rate of 90.30%. Additionally, SWC, AP, and pH also significantly impacted plant community characteristics in alpine grassland (*p* < 0.05), while other factors showed no significant effects (*p* > 0.05).

## 4. Discussion

### 4.1. Effects of Topography on Plant Community Characteristics in Alpine Grassland

Variations in ecological factors such as light, temperature, moisture, and nutrients under slope aspect and slope position conditions contribute to the diversity of plant species community distribution patterns and community types in grassland plant communities [23]. Plants can effectively exploit the subtle resource heterogeneity among environments, thereby occupying distinct ecological niches and facilitating coexistence among species [24]. Analysis of family, genus, and species composition (Table 2) reveals that plant species in the six types of topographies of alpine grassland communities studied were more abundant on the shady slope than on the sunny slope. The species were predominantly found in the families Poaceae, Leguminosae, Compositae, Rosaceae, Ranunculaceae, Polygonaceae, and Cyperaceae, with other species being rare and dispersed. Plants from Poaceae, Polygonaceae, and Cyperaceae, represented by species such as *Elymus dahuricus*, *Polygonum viviparum*, and *Kobresia humilis*, demonstrate strong environmental adaptability and are widely distributed in alpine mountainous areas. These species play a pivotal role in guiding the selection and combination of species during plant restoration and reconstruction efforts, reflecting an adaptive strategy of the plant to habitat. The heterogeneity of the environment promotes significant differentiation of plant community species in alpine grassland, leading to species migration and movement to suitable niches to better adapt to the varying water and heat distribution patterns under different environmental conditions [25]. The study of plant distribution characteristics indicates that (Table 2), BS is dominated by *Elymus dahuricus* and *Kobresia humilis*, MS by *Elymus dahuricus*, BN by *Elymus dahuricus* and *Polygonum viviparum*, MN by *Polygonum viviparum* and *Kobresia humilis*, and both TS and TN by *Polygonum viviparum*, and the distribution characteristics of the main species from seven families also vary across different topographies. It is evident that alpine grasslands located in different topographical positions on the mountain not only exhibit variations in dominant species but also significant shifts in the distribution of non-dominant species. This analysis suggests that changes in site conditions significantly impact the adaptability and ecological characteristic distribution of alpine grassland plants under varying environmental conditions, corroborating findings from previous research [26].

For alpine grasslands with relatively harsh ecological environments, the influence of topographic considerations such as slope aspect and slope position on the diversity of montane plant species is particularly significant [25]. With the change in slope aspect and slope position, the change in climate caused by hydrothermal heterogeneity in the local environment further affects the change in plant distribution pattern [27,28]. This study found that the α diversity and biomass of plants showed an upward trend from the sunny slope to the shady slope, and the difference was significant (*p* < 0.05) (Figure 2 and Figure 3), which was consistent with the research findings presented by Liu [29]. Existing studies have shown that the better the water and fertilizer conditions of the plant community, the higher its species diversity [30]. Higher light intensity on sunny slopes leads to increased surface temperature and causes fast evaporation of water in the soil, and fewer plant species can adapt to such environmental conditions and survive. In contrast, the shady slope has superior habitat and water and fertilizer resources, which can have a positive effect on the growth and development of plants, and this can result in larger individual plants, a higher α diversity index, and more biomass than the sunny slope.

There are significant differences in soil environmental factors between the bottom and top of the same slope in the research region, and these differences have an important impact on species diversity and biomass characteristics. The top and middle of the hillside have steep slopes, serious soil erosion, and a relatively harsh plant-growing environment, and fewer plant species can adapt to this environment, so the α diversity index and belowground biomass are lower. The growth environment at the bottom is just the opposite, which finally contributed to the significant optimization of the characteristics of total plant cover, average plant height, α diversity index, and belowground biomass; this is compatible with Ru’s [31] research findings on grassland plants in the loess hilly terrain. In addition, this study also found that the order of aboveground biomass on sunny slopes at different slope positions was opposite to that on shady slopes (Figure 3); this outcome is compatible with the study conclusions of Ma and Cui [32,33], but is contrary to the research result of Ru [34]. On the one hand, it is due to the different habitats formed by the differences in topographic conditions in the research region; additionally, it may be influenced by human disturbance factors, resulting in a more complicated link between the changing trend of aboveground biomass, slope aspect, and slope position, which may be investigated further later. The formation process of alpine grassland plant communities has an obvious environmental screening effect on topographic gradients [35], and the existence of an environmental screening effect makes the species living under different environmental conditions often show consistent adaptive traits [36]. This further proves that following the mutual adaptation mechanism of plants and the environment, knowing the function of environmental screening in the formation of plant communities has important guiding significance for plant restoration and reconstruction in this area.

### 4.2. Effects of Topography on Soil Factors in Alpine Grassland

Under topographic conditions, differences in plant composition and productivity lead to a heterogeneous distribution of soil properties. During long-term operation, such variations are primarily attributed to climatic differences induced by topographic factors such as slope aspect and position [37], with topographic and climatic environments typically associated with variations in soil moisture and erosion potential, which can also effectively explain the spatial distribution differences in plant communities [38]. In the Northern Hemisphere, the sunny slope (southern slope) shows the characteristics of strong light, high temperature, and large water evaporation due to the most solar radiation, whereas the shady slope (northern slope) is limited by solar radiation, resulting in a relatively cold and humid environment [39,40]. Previous studies have shown that a humid and cold environment is conducive to the accumulation of soil moisture and nutrients [41], aligning with the findings of this study that changes in slope aspect from sunny to shady slopes result in differences and incremental changes in soil moisture and nutrient contents (Figure 4 and Figure 5). On one hand, due to high plant coverage, species diversity, and biomass on shady slopes, soil C, N, and P accumulate significantly through the plant–litterfall–soil–microbial system [42]. On the other hand, the shaded slope has low temperature and high soil water content, leading to weak soil microbial activity, which slows the mineralization rate and delays the breakdown of organic matter, creating favorable conditions for nutrient storage in the soil [43]. Additionally, studies have suggested that steeper slopes tend to have lower soil nutrient content and are more prone to nutrient loss [44]. In this study, the slope of the sunny slope (average 27°) was steeper than that of the shady slope (average 21°) (Table 1), resulting in richer nutrient accumulation and better nutrient retention and soil fertility maintenance on the shady slope.

Significant variances in soil moisture and nutrient content influenced by slope position changes were observed (Figure 4 and Figure 5), consistent with prior research [34,45]. Differences in soil moisture and nutrient content across different slope positions are primarily due to soil properties and the redistribution of surface soil by runoff [46]. In the study area, the upper part of the slope is high and steep, with higher potential evapotranspiration, rapid water drainage during rainfall, and stronger leaching by surface runoff and water gravity [47], leading to downward loss of soil C, N, and P elements, resulting in lower soil nutrient content and higher pH. Conversely, nutrient accumulation is strongest at the bottom, where gravity and leaching wash nutrients downward, resulting in higher C, N, and P contents. Meanwhile, this study also demonstrated that variations in soil C, N, and P contents correlate with plant diversity and biomass (Figure 7), similar to Dufeng’s findings [48]. In this study area, the bottom plants are tall, plant diversity is rich, and growth is lush, with better-developed soil that has greater viscosity and erosion resistance than the middle and top, facilitating nutrient enrichment [49]. Additionally, heavy rainfall in the rainy season creates substantial surface runoff and soil water flow; the gentle slope at the bottom easily retains water, capturing nutrients flowing down from the top [34]. In turn, higher soil moisture content enhances enzyme activity, inducing mineralization and releasing more available nutrients [50]. Thus, precipitation-induced surface runoff shapes the soil environment, leading to variations in soil nutrient content across slope positions.

The content of soil C and N in the study area exhibits a decreasing trend in vertical spatial distribution, with noticeable “surface aggregation” (Figure 5 and Figure 6), as the organic matter decomposed and synthesized by litter provides the main source of C and N for the soil. Initially, organic matter accumulates in the surface soil and then migrates and diffuses to deeper layers under the action of water or other media, forming a distribution pattern where soil C and N content gradually decreases from the surface to deeper layers [51]. This study further verifies this phenomenon. Compared to C and N, the spatial variability of soil P content is smaller (Figure 5 and Figure 6) because P is a sedimentary element, primarily derived from long-term rock weathering, and has low mobility, resulting in minimal differences between soil layers from 0 to 100 cm, thus the spatial distribution of soil P content in different soil layers remains relatively stable [52]. The soil pH is slightly acidic across all six types of topography soils and shows a weak tendency to increase with soil layer depth (Table 4), due to salt-based ions being primarily leached from the upper soil layer and aggregated in the lower soil layer [53], resulting in a slightly lower pH in the upper soil layer than in the lower soil layer.

### 4.3. Relationship Between Plant Community Characteristics and Soil Factors

Environmental factors significantly influence the composition, structure, function, distribution, and biomass of plant communities within grassland ecosystems [54]. As a key environmental factor for plant survival, soil can profoundly affect the shaping of plant communities through the “mold effect” [55]. In turn, the growth of plants impacts the accumulation of soil nutrients, establishing an interactive relationship of interconnection and mutual influence between plant community characteristics and soil environmental factors, together shaping the uniquely ecological characteristics of grassland ecosystems [34]. As the topographic gradient changes, the interaction of soil factors directly or indirectly alters the features of plant communities in the region. The results of correlation analysis in this paper showed that SWC, SOC, TN, and pH had a significant effect on *Ma*, *P*, *H*′ index, and biomass (*p* < 0.05), indicating that these four soil factors were the key factors limiting plant growth in the study area (Figure 7). This finding aligns with the research of Ma and Zhang [33,41], further emphasizing the driving role of SWC, SOC, TN, and pH on plant community characteristics. According to the results of redundancy analysis (RDA) (Figure 8), SOC, TN, SWC, AP, and pH had a significant effect (*p* < 0.05) on the characteristics of the vegetation community, and it can be inferred that the characteristics of the vegetation community are mainly affected by the above five soil factors, of which SOC is the key influence factor. This is because carbon serves as the framework element of plants, and changes in soil carbon content will have a significant impact on plant diversity and biomass, consistent with the findings of Yang and Xiang [52,56]. The study area is a crucial zone for the summer activities of herdsmen’s livestock, and the presence of cattle, sheep, and horse entities, along with their dung, was found during the investigation, not excluding that the activities of large herbivores can increase the accumulation of soil carbon in fragile mountain ecosystems [57]. Additionally, the climatic conditions of low temperatures and hypoxia at high elevations may also contribute to higher soil carbon content [58]. The results of both correlation analysis and redundancy analysis are consistent, indicating that SOC, SWC, TN, and pH are the dominant factors influencing plant community characteristics in alpine grassland. Given the complex topography and landforms of the area where the alpine grassland plant is located and the interference of multiple factors, it is forced to continuously adjust its resource utilization strategies and genetic characteristics that match, and the natural restoration process is relatively slow. Therefore, achieving efficient ecological restoration requires a thorough understanding of the habitat conditions, community composition, and diversity features involved in plant restoration processes. By meeting the habitat requirements of different species, it is possible not only to improve the survival rate of community species but also to enhance their adaptability to external disturbances, thereby accelerating the recovery and succession process of herbaceous plants in alpine mountainous areas.

## 5. Conclusions

Slope aspect and position, and their interaction, significantly influence the characteristics of plant communities in alpine grasslands of mountainous areas, primarily achieved by directly affecting soil factors (soil moisture and nutrient content). The characteristics of plant communities and soil factors exhibit concurrent variations along the slope aspect and slope position gradient; the change trend is shady slope > sunny slope, bottom > middle and top. Additionally, the phenomenon of “enrichment” of soil moisture and nutrient content is pronounced at the bottom of slopes, showing a decreasing trend in vertical spatial distribution with notable “surface aggregation”. There is a strong correlation between soil factors and plant community characteristics, with SOC, SWC, TN, and pH identified as the most critical factors influencing plant community changes along the slope aspect and slope position gradient. SOC, SWC, and TN act as promoting factors for plant communities, whereas pH serves as a restrictive factor. SOC is a decisive soil environmental factor contributing to plant community differentiation along the slope aspect and slope position gradients.

From the current perspective, it is essential to properly manage the number of livestock as well as the frequency and intensity of human disturbances in the alpine grassland areas of Tulugou National Forest Park. In the long term, the restoration of degraded plant communities should adopt tailored measures for different terrains and plant components. Moreover, this study focused solely on the influence of main soil nutrients and water factors on the differentiation of plant community characteristics in alpine grassland from aspects of slope and position latitude. Future research should explore the distribution patterns of plant communities across different topographic dimensions and their relationships with environmental factors such as climate, soil, and topography, as well as the driving mechanisms of microorganisms. Continuous and in-depth research will provide a theoretical basis for biodiversity conservation, restoration of degraded ecosystems, and informed decision-making and management by regional governments.

## Figures and Tables

**Table 1:** Basic overview of the sample plots.

Slope Aspect	Slope Position	Elevation/m	Slope Degree/(°)
Sunny slope (South-facing)	Bottom	3070	21
	Middle	3170	26
	Top	3270	33
Shady slope (North-facing)	Bottom	3070	17
	Middle	3170	21
	Top	3270	26

**Table 2:** Plant distribution characteristics of different topographies.

Sample Plot Types	Total Plant Coverage/%	Grass Layer Height/cm	Families, Genus, Species	Distribution of Main Species Number							Dominant Species
				Poaceae	Leguminosae	Compositae	Rosaceae	Cyperaceae	Polygonaceae	Ranunculaceae	
BS	95	15.49	13, 21, 24	4	0	1	2	2	1	1	*Elymus* *dahuricus* + *Kobresia* *humilis*
MS	93	13.27	9, 18, 21	3	0	0	1	2	2	1	*Elymus* *dahuricus*
TS	90	10.78	8, 16, 20	3	1	0	1	1	3	1	*Polygonum viviparum*
BN	98	17.38	14, 23, 27	5	1	2	1	2	1	1	*Elymus* *dahuricus* + *Polygonum viviparum*
MN	96	14.57	13, 20, 24	3	1	1	0	1	1	1	*Polygonum viviparum* + *Kobresia* *humilis*
TN	92	12.69	12, 18, 21	4	1	2	1	1	1	0	*Polygonum viviparum*

Note: BS (bottom of the sunny slope), MS (middle of the sunny slope), TS (top of the sunny slope), BN (bottom of the shady slope), MN (middle of the shady slope), and TN (top of the shady slope).

**Table 3:** Two-way analysis of variance *F* value of slope aspect, slope position, and plant community characteristics.

Factors	TC	GH	*Ma*	*P*	*H*′	*E*	AGB	BGB
Slope aspect (A)	1.78	8.51 **	17.57 **	3.17	8.32 *	20.62 **	304.53 *	1676.65 *
Slope position (P)	16.78 *	12.29 *	27.07 **	24.82 *	24.09 **	18.87	10.22 *	1039.25 **
A × P	0.11	0.82	16.55 *	19.05	12.86 *	10.77	150.64 **	98.57 **

Note: * *p* < 0.05, ** *p* < 0.01. TC (total plant coverage) and GH (grass layer height).

**Table 4:** Soil pH in different topographies.

Soil Depth/cm	BS	MS	TS	BN	MN	TN
0–10	5.52 ± 0.06 b	5.69 ± 0.08 ab	5.91 ± 0.28 a	5.53 ± 0.07 b	5.61 ± 0.11 ab	5.72 ± 0.24 ab
10–20	5.66 ± 0.05 b	5.74 ± 0.23 ab	5.72 ± 0.05 ab	5.75 ± 0.17 ab	5.76 ± 0.05 ab	5.93 ± 0.10 a
20–30	5.77 ± 0.07 ab	5.79 ± 0.10 ab	5.78 ± 0.10 ab	5.73 ± 0.12 b	5.81 ± 0.12 ab	5.95 ± 0.76 a
Average value	5.65	5.74	5.80	5.67	5.73	5.87

Note: Values represent mean ± standard deviation. Differences within the same soil depth but different topographies are represented by lowercase letters (*p* < 0.05).

**Table 5:** Two-way analysis of variance *F* value of slope aspect, slope position, and soil factors.

Factors	SWC	SOC	TN	TP	NO_3_^−^-N	NH_4_^+^-N	AP	pH
Slope aspect (A)	1.01 **	32.61 *	9.51 *	12.66	19.78	25.11	18.67	0.21
Slope position (P)	6.21 **	13.76 **	17.45 *	2.61 *	36.02 *	38.07 *	20.99	3.34
A × P	1.11	1.33	3.76	0.82	15.23 **	14.27 **	12.61 **	0.85

Note: * *p* < 0.05, ** *p* < 0.01. SWC (soil water content), SOC (soil organic carbon), TN (soil total nitrogen), TP (soil total phosphorus), NO_3_^−^-N (soil nitrate nitrogen), NH_4_^+^-N (soil ammonium nitrogen), AP (soil available phosphorus), and pH (soil pH value).

**Table 6:** Plant community characteristics of alpine grassland RDA ordination characteristic value and its explanation.

Item	Axis1	Axis2	Axis3	Axis4
Eigenvalues	0.9430	0.0327	0.0003	0.0000
Cumulative explained variation (%)	94.30	97.56	97.59	97.59
Pseudo-canonical correlation	0.9908	0.9140	0.8615	0.8314
Cumulative explained fitted variation (%)	96.63	99.97	100.00	100.00

**Table 7:** Monte Carlo test of soil environmental factors.

Soil Environmental Factors	Explains (%)	Contribution (%)	*F*	*p*
SOC	80.6	82.6	66.6	0.002
TN	7.5	7.7	9.5	0.006
SWC	2.2	2.3	3.8	0.028
AP	2.3	2.3	5.1	0.026
pH	1.7	1.7	5.0	0.026
TP	2.0	2.0	2.8	0.078
NO_3_^-^-N	0.8	0.8	2.8	0.080
NH_4_^+^-N	0.5	0.5	1.9	0.206

## Data Availability

By contacting the authors in a reasonable manner, relevant data for this study can be obtained. The data are not publicly available due to [Data are contained within the article.].
